# miR-222 is upregulated in epithelial ovarian cancer and promotes cell proliferation by downregulating P27^kip1^

**DOI:** 10.3892/ol.2013.1393

**Published:** 2013-06-13

**Authors:** CHAOYANG SUN, NA LI, BO ZHOU, ZONGYUAN YANG, DONG DING, DANHUI WENG, LI MENG, SHIXUAN WANG, JIANFENG ZHOU, DING MA, GANG CHEN

**Affiliations:** Cancer Biology Research Center, Tongji Hospital, Tongji Medical College, Huazhong University of Science and Technology, Wuhan, Hubei 430030, P.R. China

**Keywords:** epithelial ovarian cancer, miR-222, P27^Kip1^, carcinogenesis

## Abstract

Epithelial ovarian cancer (EOC) is the leading cause of female reproductive system cancer mortality in females. The majority of cases of ovarian carcinomas are not identified until a late stage. Identifying the molecular changes that occur during the development and progression of ovarian cancer is an urgent requirement. MicroRNAs (miRNAs) have been identified as gene expression regulators that induce mRNA degradation or translation blockade through pairing to the 3′ untranslated region (3-‘UTR) of the target mRNAs. In the present study, miR-222 was observed to be frequently upregulated in ovarian cancer. miR-222 upregulation induced an enhancement of ovarian cancer cell proliferation potential, possibly by downregulating its target, P27^Kip1^. A bioinformatic analysis showed that the 3′-UTR of the P27^Kip1^ mRNA contained a highly-conserved putative miR-222 binding site. Luciferase reporter assays demonstrated that P27^Kip1^ was a direct target of miR-222. Consistently, there was an inverse correlation between the P27^Kip1^ and miR-222 expression levels in the ovarian cancer cell lines and tissues. Overall, the present results suggest that miR-222 upregulation in human ovarian cancer may promote ovarian cancer cell proliferation during ovarian carcinogenesis.

## Introduction

Epithelial ovarian cancer (EOC) is the leading cause of reproductive system cancer mortality in females (1). When epithelial ovarian carcinoma is diagnosed at early stages, the survival rate is high (90%). However, the majority of cases of ovarian carcinoma are not identified until the late stage and the five-year relative survival rates for the late stage of EOC are <10% (2). Despite advances in surgery and the wide use of platinum-based chemotherapy, the survival rate of patients with late stage EOC has changed little since platinum-based treatment was introduced >30 years ago. Consequently, the identification of the molecular changes that occur during the development and progression of ovarian cancer is urgently required.

MicroRNAs (miRNAs), a class of small, non-coding RNAs, have been identified as gene expression regulators that induce mRNA degradation or a translation blockade through pairing to the 3′ untranslated region (3′-UTR) of the target mRNAs (3). There is significant evidence that the dysregulation of the miRNAs is a hallmark of cancer (4). Emerging evidence shows that miRNAs are abnormally expressed in various types of cancer and are involved in various cell functions, including tumor proliferation, drug resistance, apoptosis and metastasis. miR-222 is overexpressed in various types of tumors (5–8). miR-222 expression has been shown to induce cell growth, oncogenesis, invasion, migration and drug resistance in tumor cells (9–11), and was also reported to be a significant marker of a poor prognosis (12). However, for miR-222, the possible roles and associated target genes in ovarian cancer remain poorly elucidated. In the present study, the role of miR-222 on the carcinogenesis of ovarian cancer and the underlying mechanisms were examined.

## Materials and methods

### Human EOC tissue collection

EOC tissues were obtained from patients who had undergone surgery at the Department of Gynecological Cancer of Tongji Hospital (Huazhong University of Science and Technology, Wuhan, China), between 2009 and 2010. All patients underwent debulking and subsequently received first-line platinum/taxane-based chemotherapy. All the patients were diagnosed with EOC (stages III and IV) based on a histopathological evaluation. Informed consent was obtained from all patients. All the tissue samples were collected, immediately snap-frozen in liquid nitrogen and stored at −80°C. The tumor content of the specimens was assessed by hematoxylin and eosin staining at the Department of Pathology, Tongji Hospital. Only specimens containing >60% tumor tissue were used. This study was approved by the ethics committee of Tongji Hospital, Wuhan, China.

### Cell culture and transfection

The OV2008 and C13^*^ cells were gifts from Professor Rakesh of the Ottawa Regional Cancer Center, Ottawa, Canada. The A2780 ovarian cancer cell line was obtained from The European Collection of Cell Cultures (ECACC, Salisbury, UK). These cells were maintained in RPMI-1640 supplemented with 2 mmol/l L-glutamine and 10% fetal bovine serum (FBS). ES2, SKOV-3 and CAOV-3 were purchased from the American Type Culture Collection (ATCC) and maintained in McCoy’s 5A or Dulbecco’s modified Eagle’s medium (DMEM) containing 10% FBS. All cells were used within six months of thawing and were cultured in a humidified 5% CO_2_ incubator at 37°C. The cells were plated without antibiotics ∼24 h prior to the transfections. Transient transfections of the miRNA mimics/inhibitor (RiboBio, Guangzhou, China) were performed using Lipofectamine 2000 (Invitrogen, Carlsbad, CA, USA) according to the manufacturer’s instructions. All transfections were performed for 48 h.

### RNA extraction and qPCR

Total RNAs, including miRNAs were extracted from cultured cells or fresh ovarian cancer tissues using the GeneJET RNA Purification kit (Fermentas, Vilnius, Lithuania) according to the manufacturer’s instructions. The expression of mature miR-222 was determined with the Bulge-Loop™ miRNA qPCR Primer Set (RiboBio) with SYBR-Green qPCR; U6 snRNA was used as an internal control. P27^Kip1^ mRNA expression was analyzed with qPCR using the SYBR-Green method. All protocols were performed according to the manufacturer’s instructions and the results were normalized to the expression of GAPDH. The primer sequences were as follows: P27^Kip1^ forward, 5′-TCCGGCTAACTCTGAGGACA-3′ and reverse, 5′-AGAAGAATCGTCGGTTGCAGG-3′; GAPDH forward, 5′-AGAGGCAGGGATGATGTTCTG-3′ and reverse, 5′-GACTCATGACCA CAGTCCATGC-3′.

### Cell cycle analysis

For the cell cycle experiments, the cells were trypsinized, harvested and processed with standard methods using propidium iodide (PI) to stain cellular DNA. The cell samples were analyzed using a FACSCalibur system (BD Biosciences, San Jose, CA, USA). Histograms were analyzed for the cell cycle distribution using ModFit version 2.0 (Verity Software House, Topsham, ME, USA).

### Proliferation assays

The cells (5,000 per well) were plated in 96-well plates and grown for 96 h following transfection (final miRNA concentration of 100 nmol/l) in normal culture conditions. Cell proliferation was documented every 24 h for four days using a CCK8 assay.

### Plasmid construction and luciferase assay

The full-length 3′-UTR of P27^Kip1^ was amplified by PCR from genomic DNA of normal patients using specific primers: sense, 5′-TAAGAATATGTTTCCTTGTTTATCAGAT-3′ and anti-sense, 5′-AATAGCTATGGAAGTTTTCTTTATTGAT-3′. The amplified DNA was then cloned into psi-Check2 to generate a luciferase reporter vector. The 3′-UTR of the mutant vector of P27^Kip1^ was also constructed via the overlap extension PCR method using the following primers: sense, 5′-CTCTAAAAGCGTTGGAGCATTATGCAATTAGG-3′ and anti-sense, 5′-CCTAATTGCATAATGCTCCAACGCTTTTAGAG-3′. For the reporter assays, the cells were cultured in 24-well plates and transfected with psi-Check2-P27-3′-UTR (mutant) and miR-222 mimics (miR control). Luciferase activity was measured at 48 h post-transfection using the Dual-Luciferase^®^ reporter assay system (Promega, Madison, WI, USA) and an LB 960 Centro XS3 luminometer (Molecular Devices, Sunnyvale, CA, USA). All experiments were performed in triplicate.

### Western blotting

The cells were lysed using mammalian protein extraction reagent RIPA (Beyotime, Haimen, China) supplemented with a protease inhibitor cocktail (Roche, Mannheim, Germany). The proteins (50 *μ*g) were electrophoresed by SDS-PAGE and transferred onto PVDF membranes. Immunoblotting was performed using human anti-P27^Kip1^ antibody (1:1,000; Cell Signaling, Danvers, MA, USA), and GAPDH antibody (1:5,000) was used as a control (Cell Signaling).

### EdU incorporation and immunofluorescence microscopy

The incorporation of 5-ethynyl-2’-deoxyuridine (EdU) into actively proliferating SKOV3 cells was evaluated using a Cell-Light™ EdU Cell Proliferation Detection kit; RiboBio) following the manufacturer’s instructions. Cell immunostaining was observed with an epifluorescence microscope (Axioplan II; Carl Zeiss AG, Oberkochen, Germany) equipped with a charge-coupled device camera. Digital images were acquired and analyzed with ImageJ software (NIH, Bethesda, MD, USA).

### Statistical analysis

All values are expressed as the mean ± SD. Statistical analysis was performed with the two-tailed Student’s t-test. The Wilcoxon signed rank test was used to analyze the statistically significant upregulation of miR-222 expression in ovarian cancers. The Pearson correlation analysis was performed between the expression of miR-222 and P27^Kip1^. P<0.05 was considered to indicate a statistically significant difference in all results.

## Results

### miR-222 expression is upregulated in ovarian cancer

**Figure 1.**
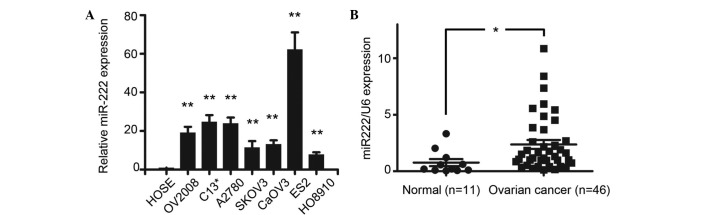
Level of miR-222 expression in ovarian cancer cell lines and tissues. (A) miR-222 expression was detected in several ovarian cancer cell lines (OV2008, C13*, A2780, SKOV3, CaOV3, ES2 and HO8910) and human ovarian surface epithelial (HOSE) cells. qPCR showed that miR-222 was overexpressed in all of the ovarian cancer cell lines compared with the HOSE cells. ^**^P<0.05 vs. HOSE cells. (B) miR-222 was detected in 11 normal ovaries and 46 ovarian cancer tissues by qPCR. miR-222 expression was significantly higher in patients with ovarian cancer compared with those with normal ovaries. ^*^P<0.05 vs. normal tissue. Values represent the mean ± SD. n=3. miRNA, microRNA.

To analyze the expression levels of miR-222, a qPCR analysis of miR-222 expression was conducted in several ovarian cancer cell lines (OV2008, C13^*^, A2780, SKOV3, CaOV3, ES2 and HO8910) and a human ovarian surface epithelial (HOSE) cell. The results showed that miR-222 was overexpressed in all ovarian cancer cell lines compared with the HOSE cells (P<0.01). Moreover, when miR-222 expression was measured in 11 normal ovarian tissues and 40 epithelial ovarian cancer tissues, qPCR showed that miR-222 was also overexpressed in tumor samples associate with the normal ovaries (P<0.05; Fig. 1A and B).

### Effect of miR-222 on ovarian cancer cell proliferation in vitro

**Figure 2.**
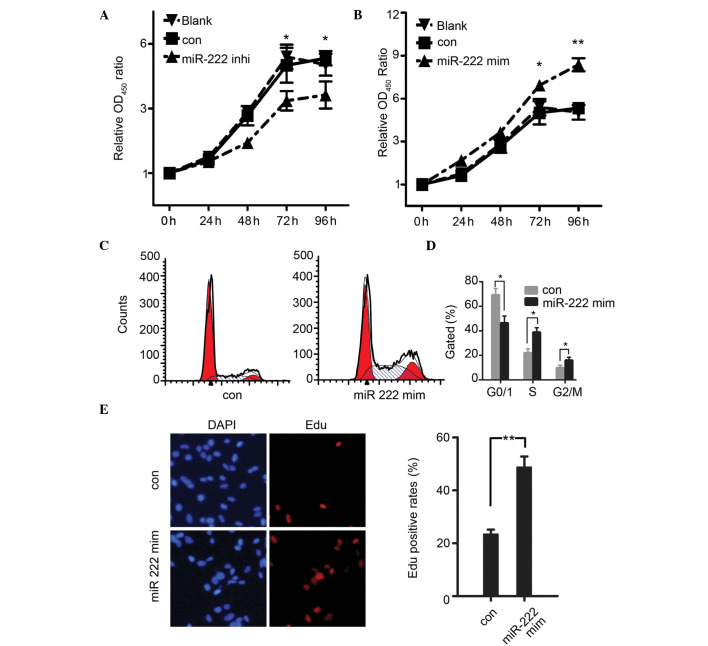
Effect of miR-222 on ovarian cancer cell proliferation *in vitro*. Cell proliferation assays were performed to evaluate the proliferation of SKOV3 cells transected with (A) miR-222 mimics or (B) inhibitors. The number of SKOV3 cells transfected with miR-222 mimics was increased compared with the cells transfected with the miR control or blank. By contrast, the SKOV3 cells transfected with miR-222 inhibitors had significantly reduced growth. (C) Cell cycle analysis showing that the SKOV3 cells transiently transfected with miR-222 mimic exhibited an increased population of cells in the S phase at 24 h post-transfection. (D) Bar chart representing the percentage of cells in the G_0_-G_1_, S or G_2_-M phases, as indicated. (E) EdU incorporation was monitored by immunofluorescence microscopy (magnification, ×100). The percentage of EdU-positive cells per optical field (at least 50 fields) was counted. Values represent the mean ± SD. n=3. ^*^P<0.05, ^**^P<0.01 vs. control. miRNA, microRNA; EdU, 5-ethynyl-2’-deoxyuridine.

To demonstrate the function of miR-222 in ovarian cancer cells, miR-222 mimics or miR-222 inhibitors were transiently transfected into the SKOV3 cells to upregulate or downregulate the expression of miR-222, respectively. qPCR confirmed the increased or decreased expression of miR-222 in the miR-222 mimics or inhibitor transfectants, respectively (data not shown). The cell proliferation assays showed that the numbers of SKOV3 cells transfected with miR-222 mimics were increased compared with the cells transfected with the miR-NC controls or blanks (Fig. 2A). However, the SKOV3 cells transfected with the miR-222 inhibitors exhibited significantly reduced growth (Fig. 2B). It is well known that enhanced cell proliferation is associated with altered cell cycles. Consequently, a cell cycle analysis was conducted in the SKOV3 cells transiently transfected with the miR-222 mimics. The results showed that the cells transfected with the miR-222 mimics exhibited an increased S phase with a decreased G_0_-G_1_ phase (Fig. 2C). The proportion of cells corresponding to the S phase was higher in the miR-222 mimic-transfected cells (38.4±4.2%) compared with the control cells (21.5±3.8%; P<0.05; Fig. 2D), while the proportion of cells corresponding to the G_0_-G_1_ phase was lower in the miR-222 mimic-transfected cells (45.9±6.2%) compared with the control cells (68.8±5.8%; P<0.05; Fig. 2D). EdU incorporation was monitored by immunofluorescence microscopy following the transfection of the SKOV3 cells with miR-222 for 48 h. A quantitative analysis of the EdU-positive cells showed that the miR-222 overexpressing cells were 49% EdU-positive compared with 24% in the miR control (>100 cells were counted). All these results indicated that miR-222 acts to promote ovarian cancer cell proliferation by increasing the population of cells in the S phase.

### miR-222 directly targets P27^Kip1^ by interacting with its 3′-UTR

**Figure 3.**
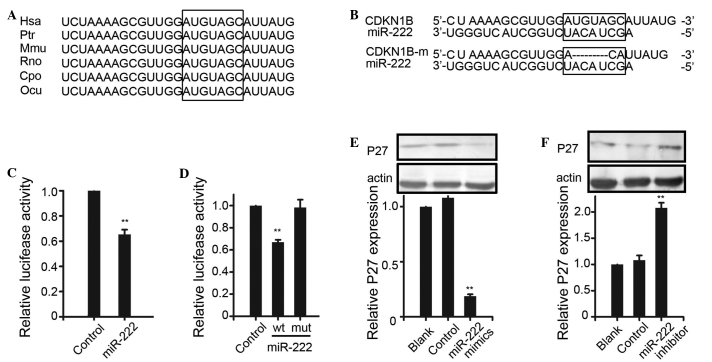
miR-222 directly targets P27^kip1^ by interacting with its 3′-UTR. (A) Predicted miR-222 binding site in P27 mRNA 3′-UTR, which is highly conserved in numerous species; the complementary sequences of the seed sequence of miR-222 are indicated. (B) Predicted binding site of the P27 mRNA 3′-UTR identified by TargetScan algorithm analysis. The mutant P27 mRNA 3′-UTR sequence is also shown. (C) Luciferase activity measured in the SKOV3 cells cotransfected with wide-type P27 3′-UTR with the miR-222 mimic or miR control. ^**^P<0.01 vs. control. (D) Luciferase activity measured in the SKOV3 cells cotransfected with wide-type or mutant P27 3′-UTR with the miR-222 mimic or miR control. ^**^P<0.01 vs. control. (E) Western blotting demonstrating that the miR-222 mimic downregulated P27 expression in the SKOV3 cells. ^**^P<0.01 vs. control or blank. (F) By contrast, the miR-222 inhibitor markedly increased the expression of P27 in the SKOV3 cells. ^**^P<0.05 vs. control or blank. Values represent the mean ± SD, n=3. ^**^P<0.01 vs. control or blank. 3′-UTR, 3′ untranslated region; miRNA, microRNA.

To further investigate the molecular mechanism by which miR-222 promotes ovarian cancer proliferation, a TargetScan algorithm analysis was performed to identify the putative downstream target genes of miR-222, particularly those with tumor suppressive effects and the ability to regulate the cell cycle to promote tumor proliferation. Based on this rationale, CDNK1B (encoding protein P27^Kip1^), a tumor suppressive gene critical for cell cycle regulation, was selected. As shown in Fig. 3A, the 3′-UTR of the P27^Kip1^ mRNA has a single predicted binding site that is highly conserved in numerous species. To investigate whether miR-222 is able to directly target P27^Kip1^ by interacting with its 3′-UTR *in vitro*, the wild-type 3′-UTRs of P27^Kip1^ were cloned and inserted downstream of a luciferase reporter gene (Fig. 3B). Subsequently, the miR-222 mimics or miR controls were cotransfected with wild-type 3′-UTR reporter vectors into the SKOV3 cells. It was observed that the miR-222 mimics decreased the relative luciferase activity of the wild-type 3′-UTR reporter vector by 37% (Fig. 3C). Furthermore, the predicted binding site of miR-222 in the 3′-UTR was mutated using overlapping PCR (Fig. 3B). Luciferase reporter assays showed that the mutant 3′-UTRs of the P27^Kip1^ vector abrogated the repression of luciferase activity caused by miR-222 overexpression, indicating that such regulation was dependent on a specific sequence (Fig. 3D). Next, the present study investigated whether miR-222 was able to regulate the expression of the P27^Kip1^ protein levels. A western blot analysis showed that the enforced expression of miR-222 significantly repressed endogenous P27^Kip1^ expression compared with the cells transfected with the negative control and blank (Fig. 3E). By contrast, following transfection with the miR-222 inhibitor in the SKOV3 cells, the expression of P27^Kip1^ was clearly increased compared with the cells transfected with the negative control or blank (Fig. 3F). Together, these data indicate that P27^Kip1^ protein expression is regulated by miR-222 in ovarian cancer.

### Expression levels of miR-222 and P27^Kip1^ are inversely correlated in patients with ovarian cancer

**Figure 4.**
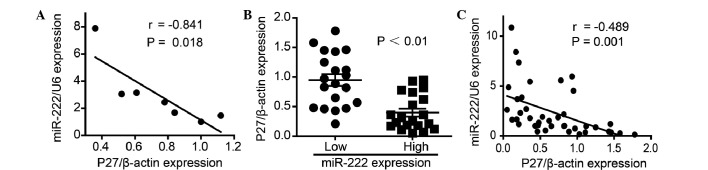
Expression of miR-222 and P27 are inversely correlated in patients with ovarian cancer. (A) miR-222 expression was determined by qPCR in seven advanced ovarian cancer cell lines. The association between P27 and miR-222 expression was detected by a Pearson correlation analysis (r=−0.841, P=0.018). (B) Patients with high miR-222 expression exhibited significantly lower P27 mRNA expression compared with the patients with low miR-222 expression (P<0.01). (C) Expression values of all 40 patients were plotted on a graph and an inverse correlation that was statistically significant was observed between P27 and miR-222 expression (r=−0.489, P=0.001). miRNA, microRNA.

P27^Kip1^ mRNA expression was first evaluated using qPCR in the ovarian cancer cells to determine the association between the expression of P27^Kip1^ and miR-222. Just as P27^Kip1^ was demonstrated to be a target gene of miR-222, an inverse correlation was observed between P27^Kip1^ and miR-222 in the ovarian cancer cells (r=−0.841, P=0.018; Fig. 4A). Furthermore, qPCR was performed in 40 ovarian cancer samples. The data showed that P27^Kip1^ expression varied between carcinomas, and that when patients were divided into two groups according to miR-222 expression, the patients with high miR-222 expression levels presented with significantly lower P27^Kip1^ mRNA expression compared with patients with low miR-222 expression levels (P<0.01; Fig. 4B). Moreover, a Pearson correlation analysis of these patients revealed a significant inverse correlation between P27^Kip1^ and miR-222 expression (r=−0.489, P=0.001; Fig. 4C). Therefore, P27^Kip1^ mRNA expression was associated with the expression of miR-222, suggesting that miR-222 was able to negatively regulate P27^Kip1^ expression in human ovarian cancer.

## Discussion

P27^Kip1^, encoded by the CDKN1B gene, is a member of the Cip/Kip family of cyclin-dependent kinase inhibitors that function to negatively control cell cycle progression. P27^Kip1^ binds to CDK2 and cyclin E complexes to prevent cell-cycle progression from the G_1_ to S phase. P27^Kip1^ also acts as a tumor suppressor, and its expression is often disrupted in human cancers (13), indicating that its levels of expression have prognostic and potentially therapeutic implications. In ovarian cancer, decreased P27^Kip1^ levels have been correlated with tumor grade, chemotherapy resistance and poor patient survival (14–16). P27^Kip1^ dysfunction is generally regarded as being due to gene mutation or loss of heterozygosity of P27, but it would be of great interest to study other potential post-transcriptional mechanisms such as miRNA-dependent translation suppression.

miR-222 has been shown to be overexpressed in various types of cancer, such as pancreatic cancer, papillary thyroid carcinoma, glioblastoma and prostate carcinoma (5,17–19), and has been reported to an important marker of poor prognosis in ovarian cancers (14–15). In the present study, it was observed that miR-222 was frequently upregulated in ovarian cancer. Furthermore, the results also provided functional evidence concerning the possible role of miR-222 in ovarian cancer, as it was demonstrated that miR-222 upregulation is able to induce an enhancement of ovarian cancer cell proliferation potential, possibly by downregulating its target, P27^Kip1^. A bioinformatic analysis showed that the 3′-UTR of the P27^Kip1^ mRNA contained the highly-conserved putative miR-222 binding site. A luciferase reporter assay demonstrated that P27^Kip1^ was a direct target of miR-222. Consistently, there was an inverse correlation between the P27^Kip1^ and miR-222 expression levels in the ovarian cancer cell lines and tissues. Overall, the present results suggest that miR-222 upregulation in human ovarian cancer may promote ovarian cancer cell proliferation during ovarian carcinogenesis. We propose that this fine-tuning regulatory action exerted by miR-222 on the levels of P27^Kip1^ protein present in the ovarian cancer cells may be considered as a sophisticated mechanism, which ensures a rapid response in P27^Kip1^ levels to any environmental and intracellular variations.

In conclusion, the present study suggests that the over-expression of miR-222 may contribute to the growth and progression of ovarian cancer, at least in part by repressing P27^Kip1^ expression. Additional functional studies are now required to aid in our comprehension of the molecular basis of the formation of this carcinoma, and to provide new evidence for developing innovative therapies targeting the specific molecular mechanisms of ovarian cancer.
